# Cellulose long fibers fabricated from cellulose nanofibers and its strong and tough characteristics

**DOI:** 10.1038/s41598-017-17713-3

**Published:** 2017-12-15

**Authors:** Abdullahil Kafy, Hyun Chan Kim, Lindong Zhai, Jung Woong Kim, Le Van Hai, Tae June Kang, Jaehwan kim

**Affiliations:** 1 Creative Research Center for Nanocellulose Future Composites, Department of Mechanical Engineering, Inha University, Incheon, 222212 Republic of Korea; 2 Advanced Materials Laboratory, Department of Mechanical Engineering, Inha University, Incheon, 222212 Republic of Korea

## Abstract

Cellulose nanofiber (CNF) with high crystallinity has great mechanical stiffness and strength. However, its length is too short to be used for fibers of environmentally friendly structural composites. This paper presents a fabrication process of cellulose long fiber from CNF suspension by spinning, stretching and drying. Isolation of CNF from the hardwood pulp is done by using (2, 2, 6, 6-tetramethylpiperidine-1-yl) oxidanyl (TEMPO) oxidation. The effect of spinning speed and stretching ratio on mechanical properties of the fabricated fibers are investigated. The modulus of the fabricated fibers increases with the spinning speed as well as the stretching ratio because of the orientation of CNFs. The fabricated long fiber exhibits the maximum tensile modulus of 23.9 GPa with the maximum tensile strength of 383.3 MPa. Moreover, the fabricated long fiber exhibits high strain at break, which indicates high toughness. The results indicate that strong and tough cellulose long fiber can be produced by using ionic crosslinking, controlling spinning speed, stretching and drying.

## Introduction

Cellulose is the most abundant raw material on earth. About billion tons of cellulose is being produced from various plants every year. Cellulose is renewable, biodegradable, cheap, thermally stable, light in weight and have many other good properties. Plant cellulose fibers are made of highly crystalline microfibrils, so-called cellulose nanofiber (CNF), which have unique properties and sizes^1–3^. Recently, cellulose has received huge attention because of its broad impact in many areas such as pharmaceuticals, coatings, food, textiles, laminates, sensors, actuators, flexible electronics and flexible displays^4–11^. CNF has typical width around 5~20 nm and a length of several micrometers. CNF can be isolated by homogenization, grinding, micro-fluidization, acid hydrolysis, and oxidation^12–19^. CNF is not soluble in water but highly dispersible which makes it possible to make a suspension with a certain viscosity. This indicates that it is possible to spin CNF suspension. CNF has anisotropic physical properties. The elastic modulus of highly crystalline CNF was reported about 150 GPa in the longitudinal and about 18–50 GPa in the transverse direction^20,21^.

Although CNF has superior mechanical properties, it is too short to be used for fibers of strong and environmentally friendly composites, for example, CNF fiber reinforced composites. Thus, fabrication of a cellulose long fiber with CNF is challenging without sacrificing high mechanical properties of CNF. Generally, alignment of the nanofibers can improve the mechanical properties^22^. Many attempts were already reported to align CNF such as magnetic field, electric field, shear force and mechanical stretching^23–27^. CNF orientation using shear force is very simple and have a potential for large scale production. Spinning is a promising and efficient way to get uniaxial oriented CNFs. Besides spinning, mechanical stretching can also improve the CNF orientation resulting in high mechanical properties^28^.

This paper aims at developing cellulose long fiber by spinning CNF suspension followed by stretching. In this study, CNF was isolated from hard wood pulp using (2, 2, 6, 6-tetramethylpiperidine-1-yl) oxidanyl (TEMPO) mediated oxidation. CNF suspension was spun in CaCl_2_ solution to form wet fibers with different spinning speed. To improve the mechanical properties, stretching and drying were applied to wet fibers so as to form cellulose long fibers. The structure and alignment of the long fibers were investigated by field effect scanning electron microscopy (FESEM) and 2D wide angle X-ray diffraction (XRD). Successful crosslinking by Ca^2+^ ions was investigated by Fourier transform infrared spectroscopy (FTIR). Mechanical properties of the cellulose long fibers were investigated by the tensile test.

## Materials and Methods

### Materials

Hardwood pulp was received from Chungnam National University, South Korea (original source from Canada). Hardwood bleached kraft pulp (HW), in dried pad form, is a combination of Aspen and Poplar. (2, 2, 6, 6-tetramethylpiperidine-1-yl) oxidanyl (TEMPO 98%), sodium bromide (NaBr 99%), sodium hypochlorite (NaClO 15%), hydrochloric acid (HCl 37%) was purchased from Sigma-Aldrich. Sodium hydroxide anhydrous (NaOH 98%) was purchased from Daejung, South Korea. CaCl_2_ was purchased from Samchun Pure Chemical, Korea.

### Fabrication

CNF suspension was prepared from hardwood bleached kraft pulp (HW) using mild TEMPO oxidation^29–31^, aqueous counter collision (ACC). In details, HW was soaked in water for 24 hours before disintegration by a food mixer. After that, TEMPO (0.0625 g), NaBr (0.625 g) was mixed with HW. Then more water was added to make it total mass of 400 g. Then 100 ml of 15% NaClO was added with a stirrer at room temperature. The pH of the solution was maintained at 12 by adding 0.5 M NaOH. After 45 minutes, the reaction was stopped by adding 0.5 M HCl to adjust pH = 7. The oxidized cellulose was washed using deionized (DI) water using filtration. After that, oxidized cellulose was mechanically isolated using an aqueous counter collision (ACC) equipment (ACCNAC100, CNNT, South Korea) for 10 passes. The ACC equipment uses 200 MPa water jets colliding each other so as to break CNFs from pulp fibers. The widths of the nanofiber were found to be in the range of 15 to 22 nm with a length of 700 to 1000 nm. Morphology, crystallinity and thermal stability analysis of CNF suspension are included in Supplementary information S1.

**Figure 1:**
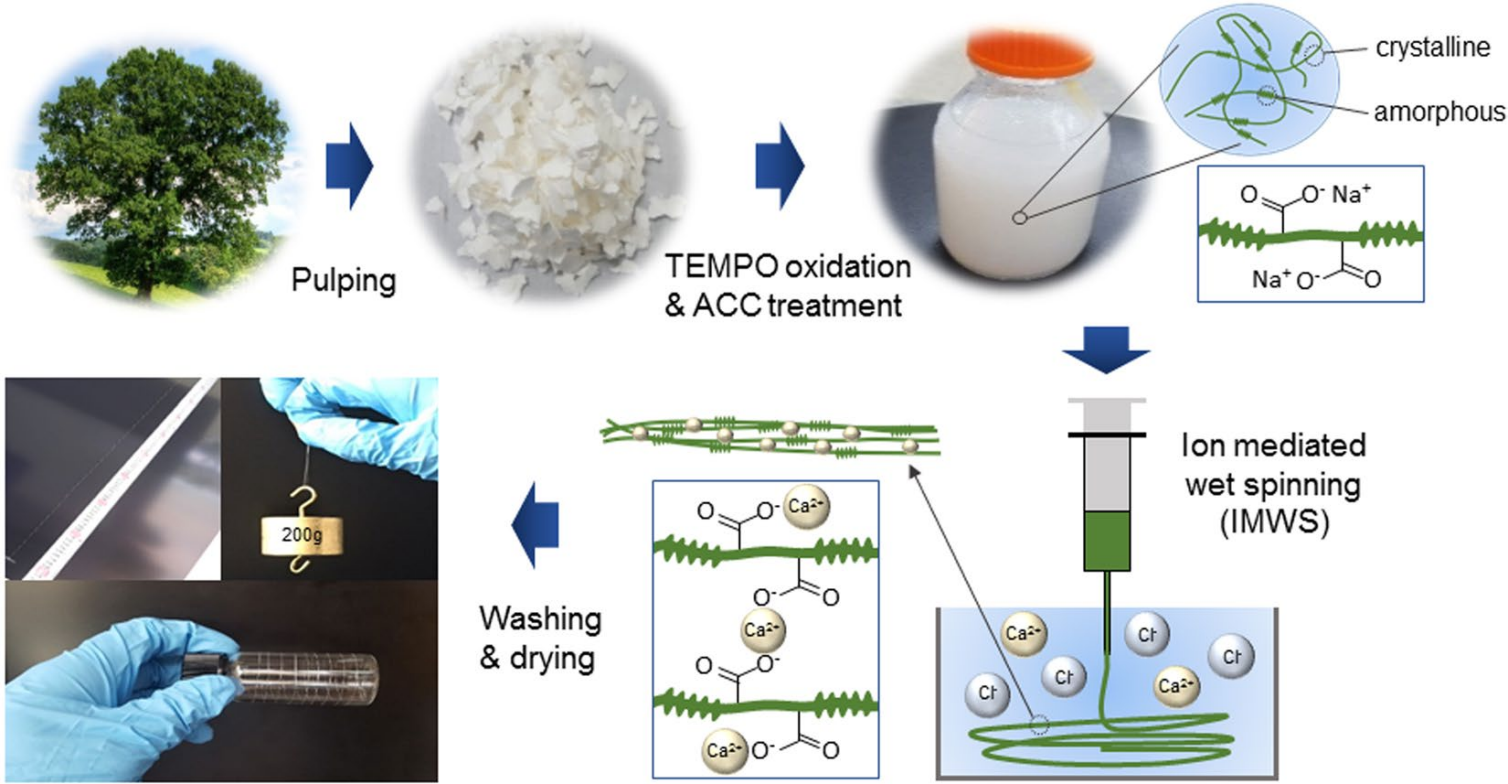
Schematic of cellulose long fiber fabrication process including spinning, crosslinking, washing, drying and stretching.

Cellulose long fiber was fabricated using ion mediated wet spinning (IMWS) in a CaCl_2_ coagulation bath. 2 wt% CNF suspension was spun in 5 wt% CaCl_2_ solution bath using a syringe pump (TJ-3A, Longer pump). The needle size was 27 G (400 µm inner diameter). The spun and wet fiber was kept in the bath for 30 minutes until the fiber is dipped into the solution after absorbing CaCl_2_. Then the spun and wet fiber was collected and dried at 60°C for 1 hour. After that, the fiber was washed with DI water to remove the loosely absorbed CaCl_2_ from the surface of the fiber and stored in DI water before drying. The wet fibers were stretched to the desired stretching ratio and dried in an ambient condition (22 °C and 25% R_H_). The schematic diagram of the fabrication process of cellulose long fiber is shown in Fig. 1. A photograph of the fabricated fiber with 1 m length is shown in the figure.

### Characterization

Morphology study was done by taking FESEM images of the fabricated fibers using Hitachi S-4300SE microscope at an acceleration voltage of 7 kV. The Energy-dispersive X-ray spectroscopy (EDX) spectra were collected using an EDAX Ametek connected with the microscope to confirm successful washing of loosely absorbed ions. The samples were prepared by coating platinum layer using an ion sputter (K575X, EMITECH).

Wide angle X-ray diffraction (WAXD) on the fabricated long fibers with different spinning speed and stretching ratio was carried out using a high-resolution X-ray diffractometer (XRD) (Bruker D8 Discover with Vantec 500 detector) at the Korea Basic Science Institute (KBSI), Daegu Center. CuKα radiation source at 40 kV and 40 mA was selected with a beam diameter of 1.0 mm in transmission mode for 2500 s at 70 mm distance from the detector. A total of 40 fibers were bundled together to get enough intensity.

To evaluate the interaction of CaCl_2_ between CNFs, FTIR was recorded by using an FTIR spectrometer (FTS 3000, BIO-RAD) in the range of 500 to 4000 cm^−1^. The samples were ground, mixed with KBr and converted into a pellet by applying pressure.

To evaluate the mechanical properties, the tensile test was done according to the standard test method, ASTM D-882-97, in the ambient condition. The gauge length of the samples were 2 cm and applied pulling rate was 0.001 mm/s. Five samples for each condition were measured and averaged the values.

### Data availability statement

The datasets generated and analyzed during the current study are available from the corresponding author on reasonable request.

## Results

### Morphology and Structure of cellulose long fibers

**Figure 2:**
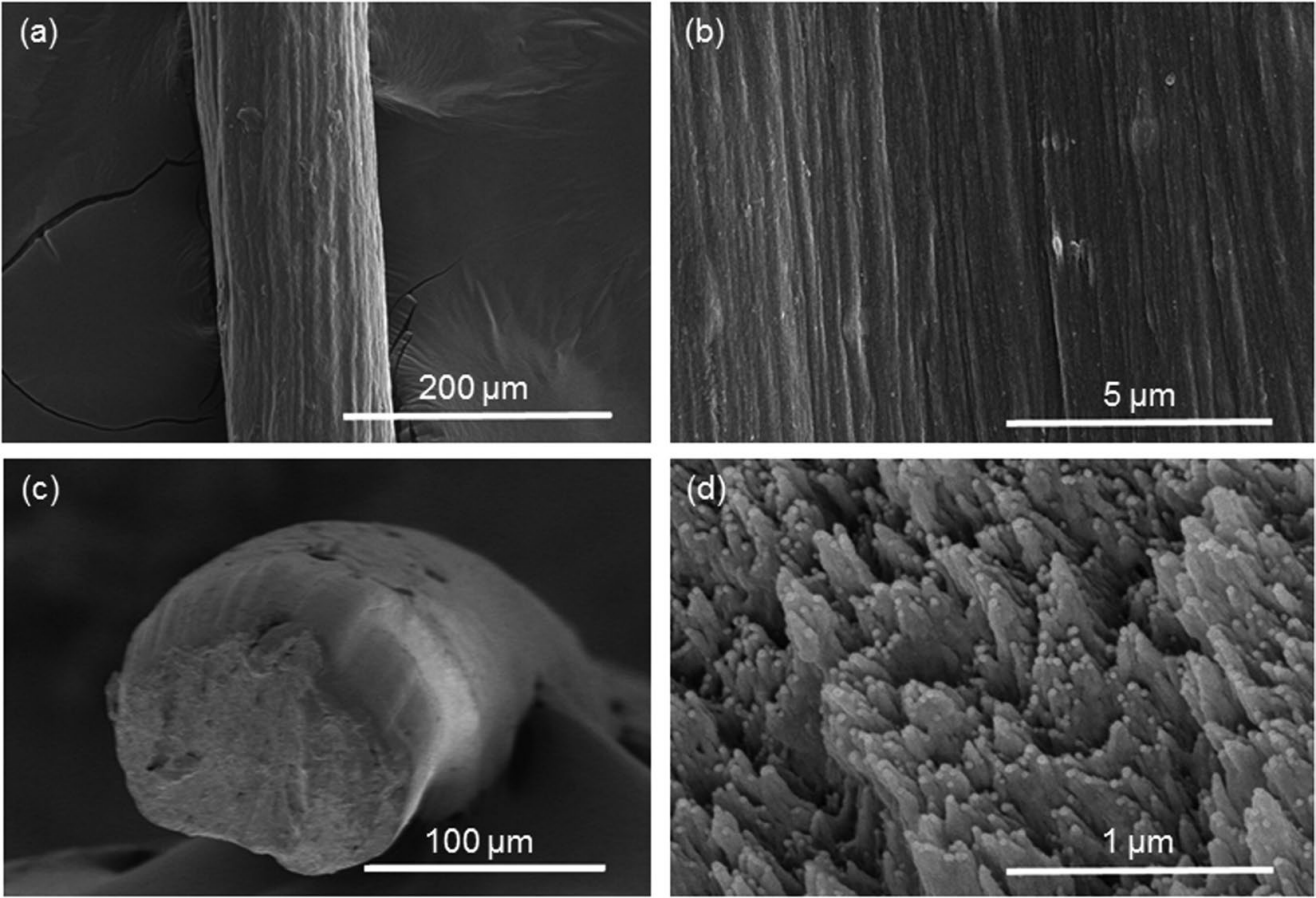
SEM Images of fabricated fibers of 2.0 ml/min spinning speed without stretching: (**a**) surface image, (**b**) surface images with different magnification, (**c**) cross-sectional image and (**d**) cross-sectional image with different magnification.

Fabrication of cellulose long fiber from hardwood CNF suspension was successfully done using CaCl_2_ coagulation bath. Spun fibers were first floating on the surface of the coagulant solution and submerged at the bottom of the solution after successful coagulation by absorbing ions. The surface and cross-section SEM images of the fabricated fibers are shown in Fig. 2 and Supplementary information S2. The surface structure does not rest on the spinning speed or stretching ratio. Individual CNFs are not clearly observed on the surface of cellulose long fibers since CNFs were crosslinked very close to each other. The cross-sections of the fabricated fibers are an almost circular shape. The shapes of all the fibers are not dependent on the spinning speed or stretching ratio. Aligned CNFs can be observed in the cross-sectional SEM image at high magnification. With stretching and high spinning speed, tightly packed long fiber can be observed.

**Figure 3:**
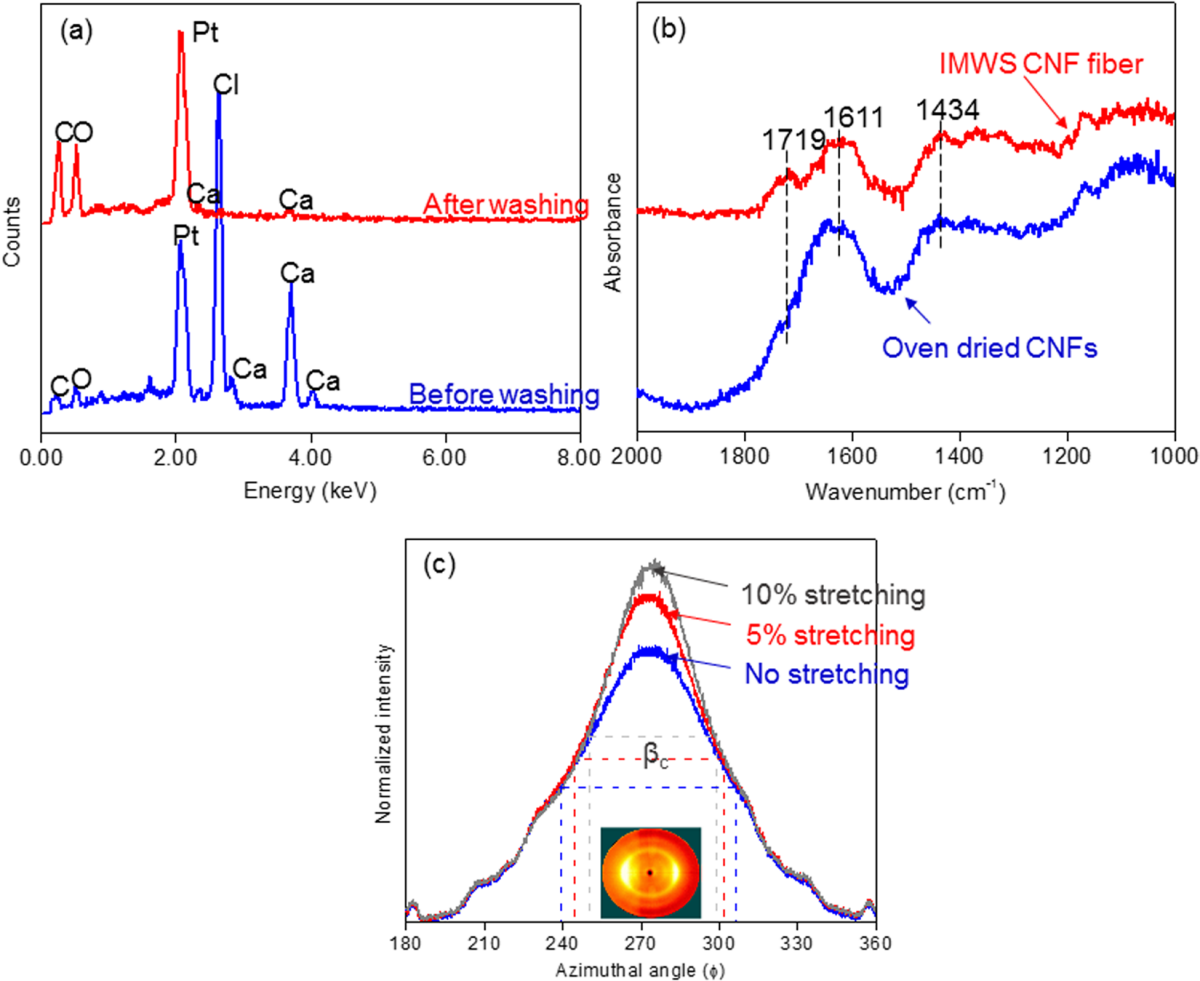
(**a**) EDX Spectra of cellulose long fiber before and after washing, (**b**) FTIR spectra of oven-dried CNFs and crosslinked long fiber and (**c**) 2D-XRD spectra of the fabricated fibers with different stretching ratios.

EDX spectra on the surface of the fabricated fiber before and after washing are shown in Fig. 3a. Before washing, lots of Ca^2+^ and Cl^−^ ions can be observed on the surface of the fiber. After successful washing, however, loosely absorbed Ca^2+^ and Cl^−^ ions were removed effectively. The amount of the element in weight percent got from EDX analysis is given in the table of Supplementary information S4.

To investigate the interaction between Ca^2+^ ions and COO^−^ functional group of cellulose, FTIR was taken and presented in Fig. 3b. FTIR was taken on the fabricated fiber and oven dried CNFs. Clear differences are shown between the oven-dried CNFs and crosslinked long fiber with respect to the characteristics and location of the bands. Asymmetric stretching mode and symmetric stretching mode for COO^-^ are observed for the oven-dried CNFs at 1611 and 1434 cm^−1^. Symmetric stretching band narrows after the ion exchange. On the other hand, broadened asymmetric stretching band was observed in the oven-dried CNFs. This broadening and narrowing of the symmetric and asymmetric stretching band of the carboxylate group can be used to identify the interaction^32–34^. Besides, the appearance of a small shoulder at 1719cm^−1^ can be observed after the ion exchange. All these changes confirmed that ions indeed offered cross-linking bridges between CNFs.

The orientation of CNFs in the fabricated fibers was evaluated using 2D-XRD. Cellulose crystal orientation symbolizes the nanofiber orientation in the fabricated fibers. 2D-XRD patterns with intensity profile vs azimuthal angle for the fibers for the spinning speed of 2.0 ml/min with different stretching ratio are shown in Fig. 3c. 2D-XRD patterns of other samples are shown in Supplementary information S3. The fabricated fibers show equilateral arcs corresponding to ($1\overline{10}$) and (200). It is known fact that the distribution along the (200) arc can be used to quantify the alignment of the nanofibers^35^. To quantify the degree of orientation, orientation index (α) of the cellulose crystals in the fabricated fibers was calculated using the following equation by azimuthal breadth analysis:

1 $${\rm{\alpha }}=({\rm{180}}-{{\rm{\beta }}}_{{\rm{c}}})/\mathrm{180}{\rm{.}}$$

Here, β_c_ can be calculated from the half width of the azimuthal direction of the equatorial reflection of (200) plane from the XRD patterns of the fiber as shown in Fig. 3c. The calculated orientation index is listed in Table 1. By increasing the spinning speed, the orientation index also increases. It can also be seen that with stretching, this value also increases. Increased spinning speed results in a high shear force on the CNF suspension so as to align CNFs. Alignment of CNFs by stretching is a very well-known phenomenon.

**Table 1:** Mechanical Properties and Orientation Index of the fabricated long fibers.

Sample Name		Orientation Index, α	Young’s Modulus (GPa)	Tensile Strength (MPa)	Strain at Break (%)
Spinning Speed	Stretching ratio				
2.0 ml/min	0	0.64	13.1 ± 0.3	224.3 ± 12.8	12.6 ± 1.0
	5%	0.68	15.9 ± 0.3	276.3 ± 10.6	8.9 ± 0.7
	10%	0.71	18.8 ± 0.5	341.5 ± 11.6	7.3 ± 0.1
5.0 ml/min	0	0.65	14.5 ± 0.2	234.6 ± 10.3	10.2 ± 0.4
	5%	0.69	17.6 ± 0.4	285.1 ± 11.1	7.1 ± 0.4
	10%	0.72	21.1 ± 0.7	383.3 ± 19.3	6.6 ± 0.4
10.0 ml/min	0	0.66	16.8 ± 0.3	249.7 ± 10.4	9.2 ± 0.4
	5%	0.70	18.6 ± 0.5	258.3 ± 10.6	6.6 ± 1.1
	10%	0.73	23.9 ± 0.2	294.1 ± 9.6	4.6 ± 0.2
Yao *et al*.^36^			22.9	357.5	2.1
Walther *et al*.^39^			22.5	275	4
Iwamoto *et al*.^37^			23.6	321	2.2

### Mechanical properties of cellulose long fibers

**Figure 4:**
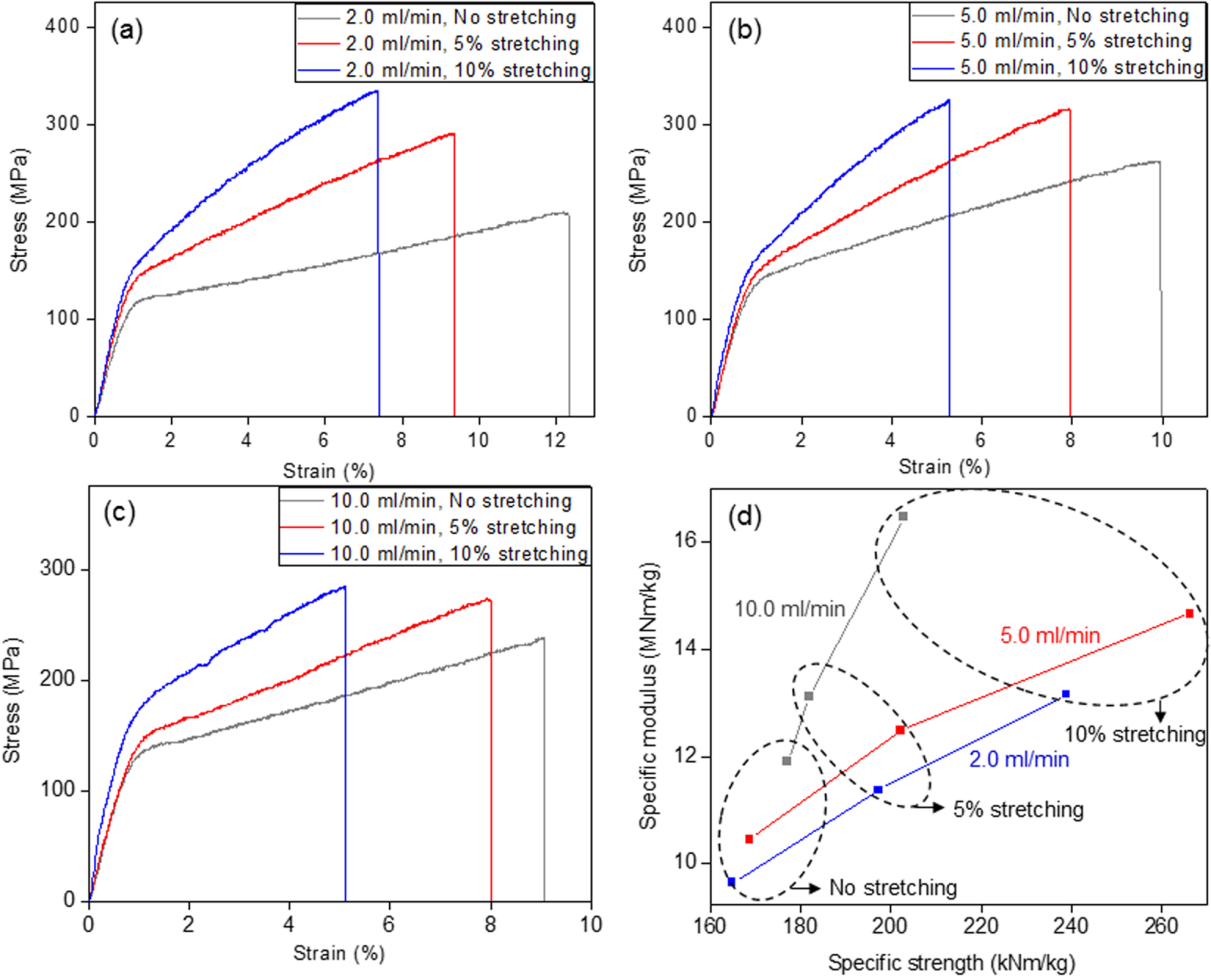
Stress-strain curves of the fabricated long fiber with different stretching ratio and spinning speed of: (**a**) 2.0 mil/min (**b**) 5.0 ml/min (**c**) 10 ml/min; (**d**) specific strength vs specific modulus curves for the fabricated long fibers.

**Figure 5:**
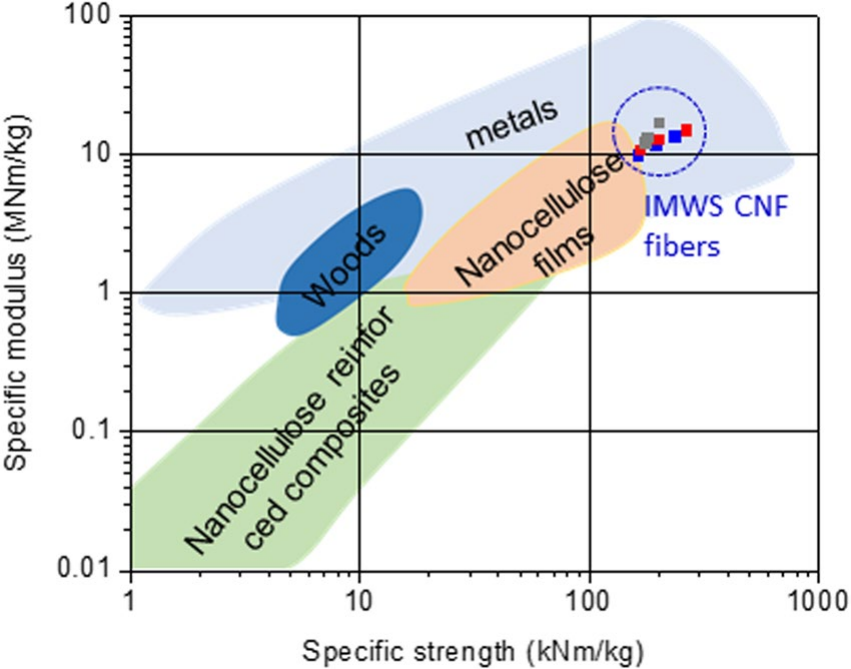
Comparison of mechanical properties of the fabricated long fibers with other materials^38^.

The mechanical properties of the fabricated long fibers from CNF suspension are presented in Table 1. The tensile modulus and strength of the fibers increased with the spinning speed. The mechanical properties improvement is associated with the increased alignment of CNFs in the fabricated fibers. Note that the elastic modulus of a single cellulose nanocrystal in longitudinal direction is about 150 GPa and in the transverse direction is in the range of 18–50 GPa^20,21^. As the orientation increases, crystals in CNF are being aligned along the fiber axis so as to increase the mechanical properties. Due to the same reason, tensile modulus and strength increased with the stretching also. For 5 ml/min spinning speed, the improvement was very high in tensile strength. In the case of 10 ml/min spinning speed, significant improvement in strength was not observed meanwhile modulus was increased. This phenomenon might be due to a fracture introduced during the stretching. As tensile modulus and strength increase, the strain at break decrease. The lowest strain at break for 10 ml/min spinning speed case proves the fact that fracture was introduced by the stretching. The tensile modulus of the fabricated long fibers ranges from 13 GPa to 23.9 GPa with the tensile strength does from 224 MPa to 383 MPa. Table 1 shows the comparison of the mechanical properties with previous studies. It can be observed that the tensile modulus and tensile strength have similar values. The fracture strain of the present fibers is highly improved over 3 times (from 2.1% (Yao *et al*.^36^) and 2.2% (Iwamoto *et al*.^37^) to 6.6%), meanwhile providing moderate improvement in tensile strength for the fiber with spinning speed of 5.0 ml/min with 10% stretching. Higher fracture strain with similar modulus and strength implies that the area under the stress-strain curve will be larger compared to lower fracture strain, ensuring the improvement in toughness of the present fibers. This improvement in terms of toughness might arise from the metal interaction (shown in Fig. 3(b)) between nanofibers in addition to hydrogen bonds. Minor effects might be caused by the use of water as a solvent in wet-spinning process. A slow evaporation of water during drying process allows a high degree of CNF alignment along the fiber length and a high packing density of CNFs on the cross section due to its relatively high surface tension and followed capillary condensation process. High toughness with the same elastic modulus means that the long fiber is not brittle and it can absorb more deformation energy than the brittle one, which is very promising for structural composites. It can be observed from Fig. 4d that with the stretching, specific strength as well as specific modulus of the fabricated long fiber also increases. Compared to the other materials shown in Fig. 5, the fabricated fibers have similar mechanical properties close to metals.

## Discussion

Combination of mild chemical (TEMPO-oxidation) and mechanical isolation of CNF provides advantages of functional groups (COO^−^Na^+^) on the surface of the nanofibers. The isolated CNFs have a high aspect ratio (length to width ratio) with a value close to 50. Presence of the functional groups makes it easy for metal interaction to produce strong and tough long fiber. Besides, chemical treatment reduces the amount of required energy for mechanical isolation methods.

From the morphology and structural study, the shape of the long fiber and the alignment of the nanofibers was quantified. The cross-section and surface morphology of the long fibers do not depend on the fabrication conditions as well as stretching ratios. The fiber shape is almost circular. EDX results (Fig. 3a and Table S4) show that there remain no sodium (Na^+^) ions after IWMS and washing. Presence of calcium ions provides an idea of replacing sodium ions in IWMS process. FTIR spectra, shown in Fig. 3b, provide the concrete evidence of strong metal interactions. From XRD patterns shown in Fig. 3c, the alignment of CNFs in long fiber was quantified. It can be easily observed that with stretching as well as with the spinning speed, the orientation was improved.

Study on mechanical properties of IWMS long fibers shows that the fibers have very high mechanical strength as well as modulus. By increasing the spinning speed as well as stretching ratio, the modulus and strength increase. The reason behind this improvement is the improved alignment of the CNFs. As alignment increases the surface contact among fibers increases which provide a better opportunity to make hydrogen bond as well as metal interactions. In the case of 10 ml/min spinning speed, the improvement in strength is not significant. The reason behind this may be due to some defects propagated during the stretching. From Table 1 it can be seen that the fracture strength reduces with the spinning speed as well as the stretching ratio. This reduction in strain may lead to the defect formations at the stretching to the fiber fabricated with 10 ml/min spinning speed. Additionally, it shows higher mechanical properties than nanocellulose films. The present fibers with high toughness indicate that they can absorb more deformation energy compared to brittle materials with similar modulus and strength. This property is very much interesting to be used for structural composites. The present fibers based on CNF building blocks have potential to be used in smart textile, structural reinforcement.

## Conclusion

Wet-spun long fiber from hardwood cellulose nanofiber was successfully fabricated by using crosslinking with Ca^2+^ ions, followed by stretching and drying. Increased spinning speed and stretching ratio improved the alignment of the cellulose nanofibers resulting in improved mechanical properties. The tensile modulus of the fabricated long fibers ranges from 13 GPa to 23.9 GPa with the tensile strength does from 224 MPa to 383 MPa, which show similar values to the previous studies. However, significant improvement in strain at break was observed compared to the previous studies. It can be said that strong and tough long fiber can be produced by using ionic crosslinking, controlling spinning speed, stretching and drying. The long fibers produced from cellulose nanofibers can be used for fiber reinforced strong and environmentally friendly composites.

## Electronic supplementary material

Supplementary Information
