# Supplementary material for: Cellulose long fibers fabricated from cellulose nanofibers and its strong and tough characteristics
Source: Sci Rep. 2017 Dec 15;7:17683. doi: 10.1038/s41598-017-17713-3 (PMC5732198; doi:10.1038/s41598-017-17713-3)
Supplement: Supplementary file 1 — Supplementary Information [file 41598_2017_17713_MOESM1_ESM.docx]

**Cellulose long fibers fabricated from cellulose nanofibers and its strong and tough characteristics**

**Abdullahil Kafy, Hyun Chan Kim, Lindong Zhai, Jung Woong Kim, Le Van Hai, Tae June Kang, Jaehwan kim^*^**

**S1 Characterization of cellulose nanofiber (CNF) from hard wood (HW):**


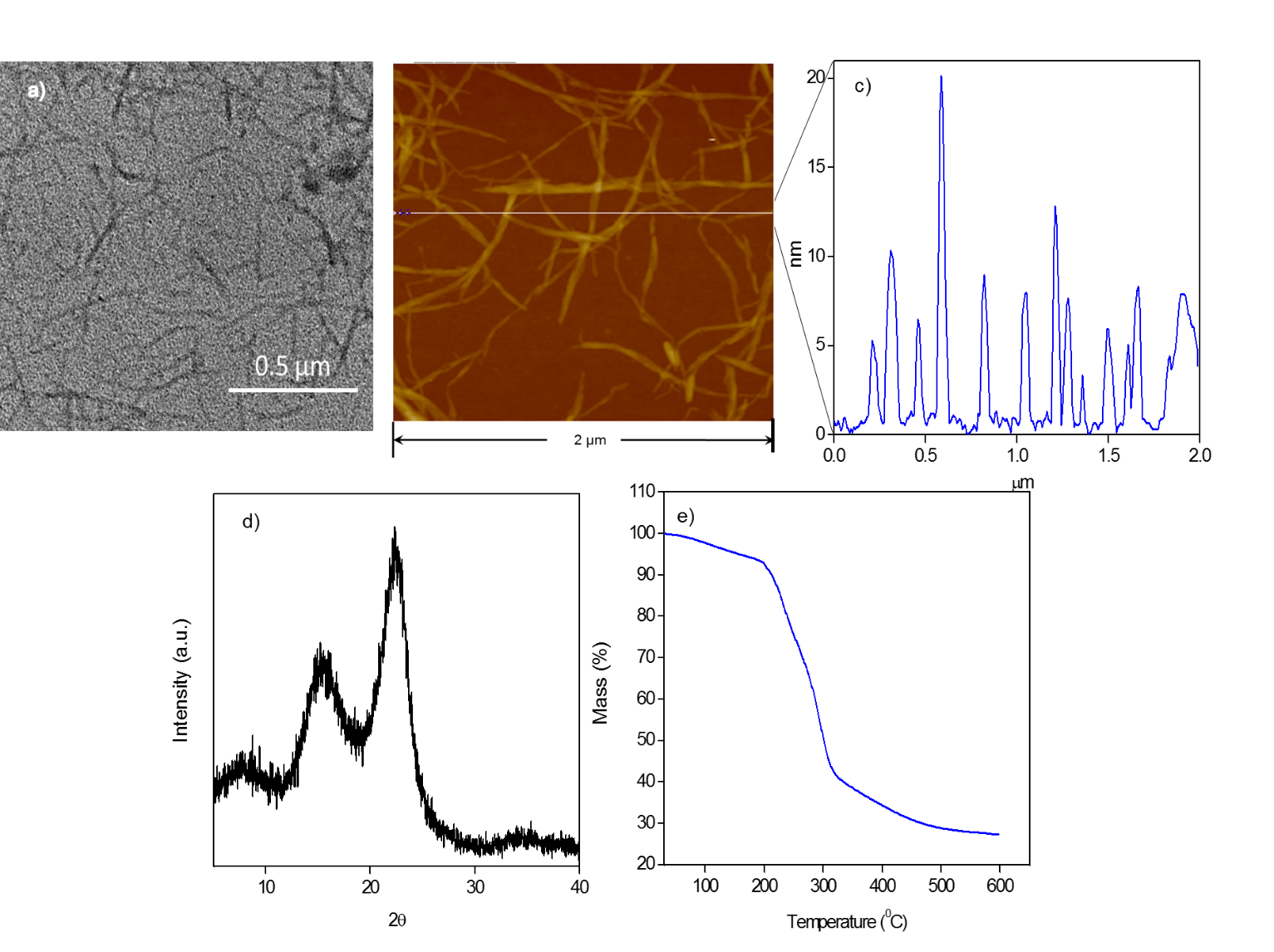


b)

Figure S1: a) TEM image b) 2D AFM image c) width of the CNFs along the white line from AFM image d) XRD spectra and e) TGA analysis of HW CNF.

The morphology and dimensions of CNF from HW was checked by using an atomic force microscopy (Veeco AFM) in tapping mode and a FE-TEM (JEOL, JEM 2100F) image are presented in Figure S1 (a-c). The dimension was measured using the imageJ V5.10 software from AFM and TEM images. The measured width was 15-22 nm with a length of 700-1000 nm.

For investigating crystallinity, XRD analysis was carried out using Rigaku RINT2000 X-ray diffractometer with wide angle goniometer. CuKα radiation source at 40 kV and 100 mA was selected with a scanning step of 0.02^º^. Calculated crystallinity index was found to be 66.3%. The XRD spectra are shown in Figure S1 (d). Following equation was used to calculate crystallinity index (CrI%):

CrI% = $\frac{(I_{200}-I_{am})}{I_{200}}\times100$ (1)

Where I_200_ is the intensity of the crystalline peak at the maximum between 2θ=22^o^ and 23^o^ and I_am_ is the intensity at the minimum between 2θ=18^o^ and 19^o^.

Thermal stability of the CNF fiber was evaluated by using a thermogravimetric dynamic thermal analyzer (NETZSCH TG 209F3) with Al_2_O_3_ crucible in an atmosphere under N_2_ gas flow. The result is presented in Figure S1 (e). The sample weight was 7 mg. Up to 200^º^C, about 7% of total mass loss was observed which may be associated with free water absorbed on the surface. At 200^º^C sudden mass loss of 60% was took place. Note that CNFs have a thermal decomposition temperature of 200^º^C.

**S2 Cross-sectional SEM images of the fabricated long fibers:**


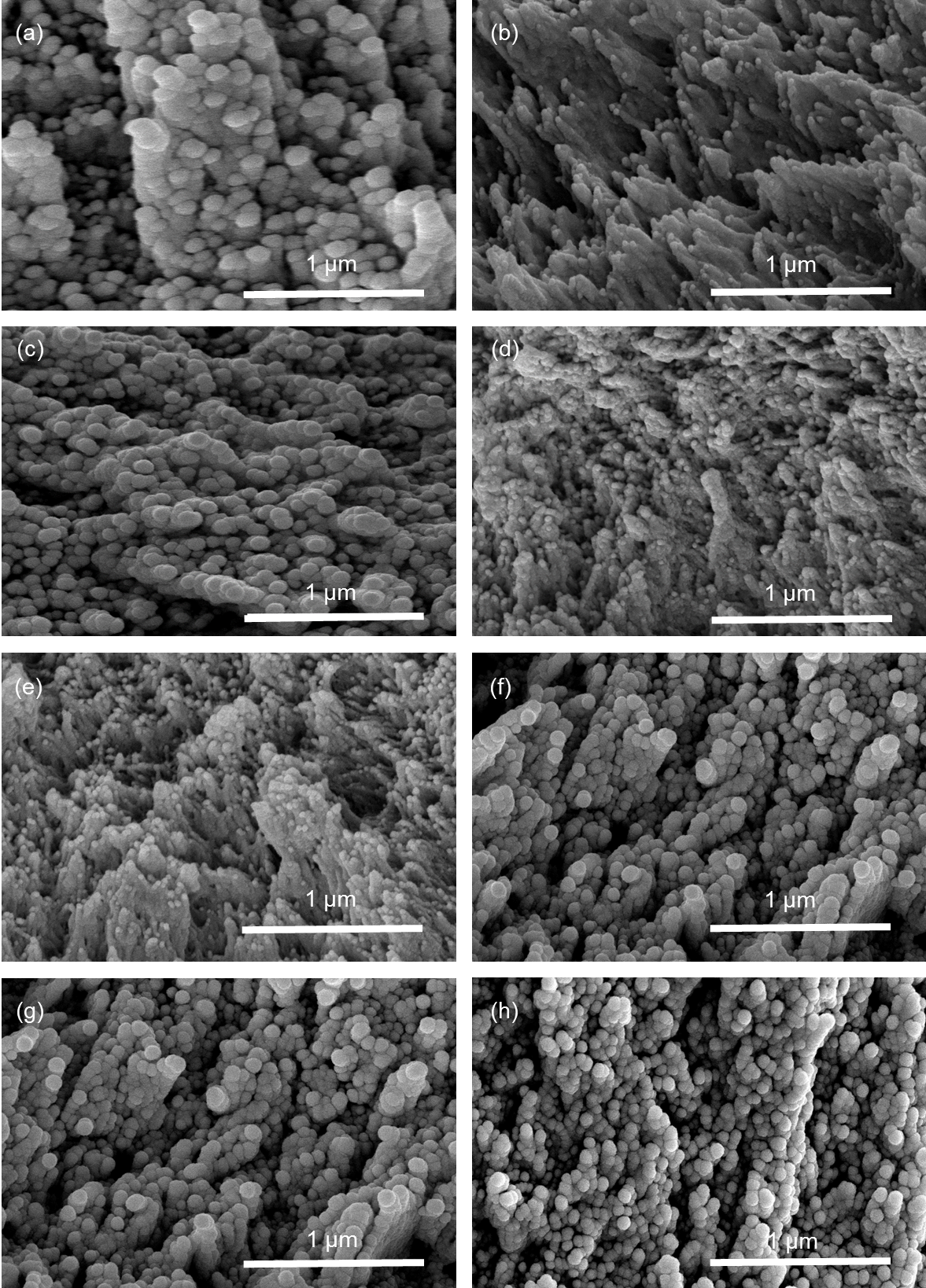


Figure S2: Cross-sectional SEM images for different conditions: a) 2.0 ml/min 5% stretching, b) 2.0 ml/min 10% stretching, c) 5.0 ml/min no-stretching, d) 5.0 ml/min 5% stretching, e) 5.0 ml/min 10% stretching, f) 10.0ml/min no-stretching, g) 10.0 ml/min 5% stretching and h)10.0 ml/min 10% stretching.

**S3 Intensity profile vs azimuthal angle from 2D XRD:**


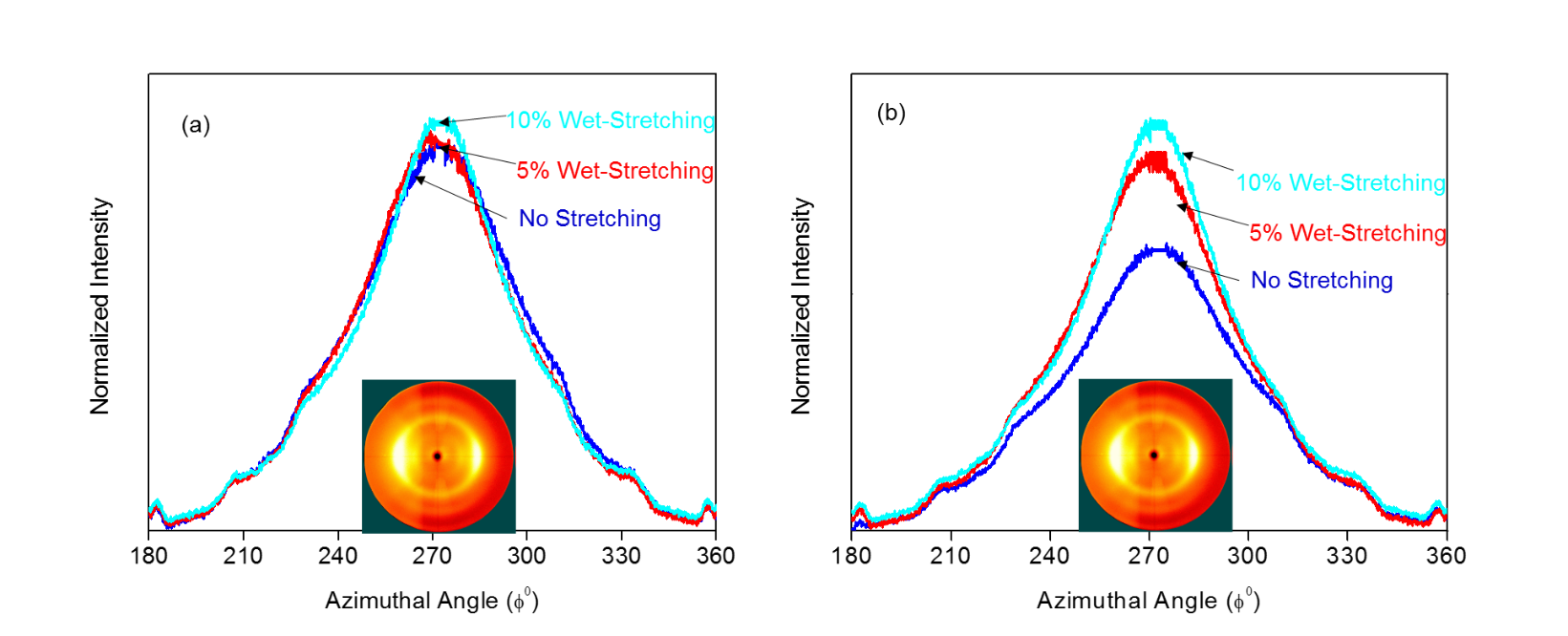


Figure S3: Intensity profile vs azimuthal angle from 2D XRD spectra: a) 5.0 ml/min and b) 10.0ml/min.

**S4 Amount of the elements from EDX analysis:**

|  | **C (wt%)** | **O (wt%)** | **Cl (wt%)** | **Ca (wt%)** |
| --- | --- | --- | --- | --- |
| Before Wash | 14.13 | 09.31 | 46.52 | 30.04 |
| After Wash | 45.45 | 48.16 | 00.00 | 06.39 |
